# Understanding the role of non-HLA antibodies in kidney transplantation: a literature review

**DOI:** 10.3389/fimmu.2026.1832945

**Published:** 2026-07-15

**Authors:** Raffin Ryeed, Eva Abubaker Sidahmed, Aleena Sajid, Sabina Al Agbar, Sonali de Chickera, Lakshman Gunaratnam, Abubaker Sidahmed

**Affiliations:** 1 Department of Pathology and Laboratory Medicine, London Health Science Centre, London, ON, Canada; 2 Department of Medicine, Division of Nephrology, McGill University Health Centre, Montreal, QC, Canada; 3 Division of Nephrology, Department of Medicine, Schulich School of Medicine and Dentistry, Western University, London, ON, Canada; 4 Department of Pathology and Laboratory Medicine, Schulich School of Medicine & Dentistry, Western University, London, ON, Canada

**Keywords:** allograft dysfunction, angiotensin type I receptor, antibody-mediated rejection, collagen, endothelin type A receptor, fibronectin, glutathione S-transferase theta 1, MHC class I chain-related molecule A

## Abstract

Non-HLA antibodies have emerged as important contributors to antibody-mediated rejection and long-term graft dysfunction in kidney transplantation, including in patients without detectable donor-specific antibodies against human leukocyte antigens (HLA-DSAs). This review summarizes current evidence on major non-HLA antibody targets, including angiotensin II type 1 receptor, endothelin A receptor, perlecan fragment LG3, vimentin, major histocompatibility complex class I chain-related molecule A, collagen, fibronectin, natural antibodies, and glutathione S-transferase theta 1, and highlights their mechanisms of formation, pathogenic roles, and clinical associations. These antibodies can arise through alloimmune or autoimmune processes, often driven by endothelial injury, ischemia-reperfusion injury, and inflammatory signaling, which may expose cryptic antigens and promote loss of self-tolerance. Mechanistically, non-HLA antibodies can mediate graft injury through complement activation, Fc receptor–dependent immune responses, and direct signaling effects on endothelial and vascular cells, leading to inflammation, microvascular injury, and fibrosis. Importantly, they can act synergistically with HLA-DSA, amplifying immune responses, and contributing to worse transplant outcomes. However, findings across studies remain inconsistent due to heterogeneity in detection methods, lack of standardized thresholds for positivity, and variability in patient populations. Additionally, temporal dynamics in antibody development and fluctuations over time suggest that static measurements may not fully capture their clinical relevance. The presence of non-HLA antibodies in healthy populations further complicates interpretation and highlights the need to better define clinically meaningful thresholds. Given the wide diversity in antigen targets, biological functions, and mechanisms of action, a one-size-fits-all approach is insufficient. Instead, individual non-HLA antibodies should be investigated through targeted mechanistic and clinical studies to clarify their specific roles in transplant outcomes. Future research should prioritize large-scale, multicenter prospective studies with standardized methodologies, alongside mechanistic investigations, to improve risk stratification and advance understanding of their impact on long-term kidney transplant success.

## Introduction

Kidney transplantation plays a vital role in improving the quality of life of individuals with end-stage renal disease and remains the most cost-effective and successful treatment option (1). With significant advancements in medicine, acute rejection rates currently stand at 15-20%, which is a substantial reduction compared to rates in the past (2). The increased success for renal transplantations can be attributed to improvements in immunosuppressant therapies, post-surgical care, and donor-recipient matching (3). Although the short-term graft survival has improved alongside acute rejection rates, long-term graft outcomes have not seen similar improvements. Thus, chronic rejection and graft failure still impact many transplant patients.

Antibody-mediated rejection (AMR) is the leading cause of graft loss after kidney transplantation (4). The criteria for an AMR diagnosis include histological evidence of microvascular inflammation (MVI), antibody interaction with the endothelium, and serological evidence of certain antibodies. Historically, AMR is known to be mediated by donor-specific antibodies against human leukocyte antigens (HLA-DSAs) circulating through the transplant recipient. These antibodies specifically target the polymorphic HLA class I and II antigens present on the donor graft that differs from the recipient. HLA-DSAs have strong associations to AMR and other poor graft outcomes in kidney transplantations (5–7). Although this is significant, patients who test negative for HLA-DSAs also experience AMR (8). Consequently, evidence has accumulated for the role of non-HLA antibodies that target antigens on the graft other than the HLA system, such as HLA-identical siblings that have experienced AMR after kidney transplantations (9). In this emerging field of transplant immunology, many studies have reported strong associations for non-HLA antibodies to be involved in the rejection and long-term survival of various solid organ transplants including the kidney, heart, and lungs (10, 11). Currently, studies on non-HLA antibodies have mixed findings regarding their clinical significance and potential pathogenic mechanisms. This review aims to summarize and evaluate recent findings of the most relevant non-HLA antibodies in the context of kidney transplantations, including their mechanisms of formation, clinical significance, pathogenic mechanisms leading to injury, and potential therapeutic avenues (**Table 1**).

**Table 1:** Summary of non-HLA antibodies in kidney transplantation and their clinical associations.

Antibody target	Antigen type	Alloantibody/autoantibody	Clinical associations
LG3 (Perlecan fragment)	ECM protein fragment	Autoantibody	AMR, DGF, vascular injury
AT1R	GPCR	Autoantibody	AMR, vascular inflammation, graft dysfunction, graft loss
ETAR	GPCR	Autoantibody	AMR, vascular rejection, reduced eGFR, graft loss
MICA	Stress-induced cell surface protein	Alloantibody	AMR, reduced graft survival, chronic graft injury
Vimentin	Intracellular cytoskeletal protein	Autoantibody	Interstitial fibrosis and tubular atrophy, graft dysfunction
Collagen (Types I, III, IV)	ECM protein	Autoantibody	AMR, transplant glomerulopathy, graft fibrosis
Fibronectin	ECM protein	Autoantibody	Transplant glomerulopathy, chronic graft injury
Natural antibodies	Polyreactive (apoptotic cells, modified self-antigens)	Autoantibody	Graft loss, TCMR, MVI
GSTT1	Intracellular enzyme	Alloantibody	AMR, graft dysfunction

AT1R, angiotensin II type 1 receptor; ETAR, endothelin A receptor; MICA, major histocompatibility complex class I chain-related molecule A; GSTT1, glutathione S-transferase theta 1; GPCR, G protein-coupled receptor; ECM, extracellular matrix; AMR, antibody-mediated rejection; DGF, delayed graft function; MVI, microvascular inflammation; TCMR, T cell-mediated rejection.

## Non-HLA antibody formation

The vasculature is the barrier between the patients’ natural immune system and the foreign organ. As such, acute vascular rejection (AVR) accounts for one of the earliest signs of complication post-renal transplantation (11). HLAs are constitutively expressed on the allograft’s endothelium cell surface, whereas non-HLA antibody production can be fueled by tissue damage (12). These non-HLA antibodies mainly recognize the antigens that are expressed by the transplanted organ’s endothelial cells to initiate the inflammatory response. Non-HLA antibodies can be categorized as alloantibodies or autoantibodies. Alloantibodies target polymorphic antigens present on the donor graft, which differ between the donor and the recipient. These include antibodies against donor specific HLA, major histocompatibility complex class I chain-related molecule A (MICA), and glutathione S-transferase theta 1 (GSTT1). Conversely, autoantibodies target self-antigens present on the donor graft and the recipient as they are not considered to be highly polymorphic in the population. Non-HLA antibodies associated with poor kidney transplantation outcomes are primarily considered to be autoantibodies capable of targeting perlecan, angiotensin type I receptor (AT1R), endothelin A receptor (ETAR), vimentin, collagen, fibronectin, and natural antibodies (Nabs). Additionally, several non-HLA antibodies can be categorized based on the nature and localization of their target antigens. G protein-coupled receptor (GPCR) autoantibodies include anti-AT1R and anti-ETAR antibodies, whereas extracellular matrix (ECM)-derived antibodies include anti-LG3, anti-collagen, and anti-fibronectin antibodies. Classically, the initiation of antibody production in response to an antigen begins with antigen presentation to T cells by antigen-presenting cells (APCs), such as dendritic cells, macrophages, and endothelial cells, either from the recipient or the donor (13). Upon recognition of the antigenic peptide presented on major histocompatibility complex (MHC) molecules, T cells become activated and provide essential help to B cells. Activated T cells express costimulatory molecules and secrete cytokines, crucial for the differentiation and maturation of antigen-specific B cells (14). Once activated by T cell help, B cells internalize the antigen through their surface immunoglobulin (Ig) receptors, process it, and present peptide fragments within MHC class II grooves to cognate T cells (15). This interaction establishes a reciprocal activation loop between T and B cells, intensifying the immune response. Subsequently, B cells undergo class-switch recombination, enabling them to produce various Ig isotypes such as IgG, IgA, or IgE, in addition to constitutively expressed IgM antibodies (16). Within germinal centers, proliferating B cells undergo somatic hypermutation, generating numerous antibody variants with diverse affinities for the antigen. High-affinity variants are selected, ultimately leading to the formation of long-term memory B cells and plasma cells capable of robust antibody production upon re-exposure to the antigen (17, 18).

### Alloantibody formation mechanisms

Non-HLA alloantibody formation arises from mismatched polymorphic antigens present in donor tissues that differ from those of the recipient. Similar to HLA antibody formation, alloimmune responses against non-HLA antigens involve antigen recognition by the recipient’s immune system through direct, indirect, semi-direct, and inverted pathways (19). In the direct pathway, intact donor APCs directly present donor antigens to recipient T cells. The indirect pathway involves recipient APCs processing and presenting donor-derived antigens to recipient T cells, critically activating CD4+ T cells and facilitating B cell differentiation into antibody-secreting plasma cells. The semi-direct pathway entails recipient APCs acquiring donor MHC molecules, enabling direct presentation of intact donor antigens. Conversely, the inverted pathway includes donor APCs presenting recipient-derived antigens, potentially influencing graft-versus-host reactions. Ultimately, these pathways lead to allo-reactive B cell activation, differentiation, and specific alloantibody production.

### Autoantibody formation mechanisms

Autoantibody formation is thought to involve endothelial injury, which may expose normally hidden cryptic or neoantigens, although multiple pathways are likely to contribute. Pre-transplant conditions such as acute kidney injury, chronic kidney disease, lupus nephritis, focal segmental glomerulosclerosis, diabetes, and preeclampsia can induce initial production of non-HLA autoantibodies (20). Post-transplant events, including ischemia-reperfusion injury (IRI), viral nephropathies like BK virus, graft pyelonephritis, acute rejection episodes, and immunosuppressive treatments, may further amplify autoantibody production *de novo*. Endothelial damage results in increased expression of self-antigens during stress conditions, potentially breaking self-tolerance and promoting autoantibody formation. Supporting this concept, Solà-Porta et al. (21) have demonstrated that the antigenic targets of non-HLA antibodies may shift over time. In particular, antibodies directed toward intracellular antigens become more prominent following transplantation.

Upon endothelial injury, autoantigens are released alongside damage-associated molecular patterns (DAMPs) and extracellular vesicles (EVs) (22). Donor kidney grafts begin releasing EVs early in the transplantation process, including during organ retrieval, preservation, and IRI, with continued release post-implantation that contributes to ongoing immune activation (23). EVs are particularly significant as they act as dynamic mediators of intercellular communication, carrying membrane-bound and intracellular autoantigens, nucleic acids, and immunoregulatory molecules that influence recipient immune responses. These vesicles interact with recipient cells through diverse ligand–receptor mechanisms and can mediate antigen presentation via direct, indirect, and semidirect pathways (24). In addition to T cell priming, EVs contribute to B cell activation and alloantibody production, as donor-derived EVs carrying HLA or non-HLA antigens can be recognized by alloreactive B cells, promoting sensitization. Furthermore, injury-associated EV subsets, such as apoptotic exosome-like vesicles, have been implicated in shaping the intragraft microenvironment by promoting tertiary lymphoid structure formation and recruiting IL-17–producing T helper 17 (Th17) cells, processes linked to chronic rejection and breakdown of B cell tolerance (25–27). These findings suggest that EVs not only serve as reservoirs of autoantigens but also actively amplify and sustain alloimmune and autoimmune responses within the graft (28). For a comprehensive overview of EV biology and their role in transplant immunology, Xu et al. (23) provide an in-depth review.

More recently, innate-like B cells (Bin cells), characterized by unique gene expression profiles including AHNAK, IL-15, and type-1 interferon, are being studied as possible contributors to local autoimmunity within kidney grafts (29). These cells produce IgG autoantibodies against renal-specific or inflammation-associated antigens, thereby bridging innate and adaptive immune responses and further promoting inflammation and graft injury.

Moreover, the inflammatory environment induced by HLA-DSAs can significantly influence autoantibody production. The inflammatory milieu fostered by DSAs can enhance the exposure of cryptic antigens and promotes epitope spreading (11). Conversely, non-HLA antibodies can potentiate HLA-driven alloimmunity by amplifying inflammation. Antibodies targeting AT1R and ETAR may be associated to proinflammatory cytokines, including TNF-α, IFN-γ, IL-1β, IL-8, and IL-17, as well as cytokines involved in B-cell activation and antibody production, such as IL-6 and GM-CSF (30, 31). These mediators upregulate endothelial HLA expression, thereby increasing antigen availability and susceptibility to HLA-DSA–mediated injury. This bidirectional amplification highlights a synergistic interplay between alloimmune and autoimmune responses. Clinically, this synergy is reflected in consistently worse transplant outcomes among patients with dual HLA-DSA and non-HLA antibody positivity compared to those with either alone, a finding that will be explored in subsequent sections. Notably, Lamarthée et al. (32) reported this interaction where HLA-DSAs and non-HLA antibodies acted as both independent and synergistic predictors of AMR, with the highest risk observed in patients harboring both antibody types. Although these key immune mechanisms occurring in both the pretransplant and posttransplant settings can theoretically promote autoantibody production, the variability and lack of consistent associations between specific autoantibodies such as anti-LG3, anti-AT1R, and anti-vimentin antibodies suggest multiple, independent immunological pathways contribute uniquely to autoantibody generation relevant in transplantation (33).

## Mechanistic action of non-HLA antibodies

Clinically, non-HLA antibodies have been implicated in acute and chronic AMR, transplant glomerulopathy, reduced graft survival, accelerated graft dysfunction, and fibrosis (20). Strong evidence exists demonstrating their clinical impact even in the absence of HLA-DSAs, highlighting their independent pathogenic potential. Despite clear clinical associations, the detailed mechanistic pathways underlying non-HLA antibody-mediated injury remain less understood. Certain non-HLA antibodies have been studied more extensively regarding their pathogenic mechanisms than others, suggesting a spectrum of understanding within this field. Accordingly, the mechanisms leading to injury and poor transplant outcomes can vary considerably based on the specific non-HLA involved. Furthermore, it is challenging to determine the isolated mechanistic effects of non-HLA antibodies because the frequent presence of HLA-DSAs often complicates interpretations. Broadly, these mechanisms can be categorized into complement-dependent and complement-independent pathways.

### Complement-dependent mechanisms

Complement activation through non-HLA antibodies typically involves IgM, IgG1, and IgG3 subclasses, which bind antigens on graft endothelial cells via their Fab regions and activate the classical complement cascade through Fc region interaction with C1q (14). Following antibody binding, C1q undergoes conformational changes, leading to sequential activation of C1r, C1s, and subsequent cleavage of C2 and C4. This forms the C3 convertase (C4b2a), promoting further cleavage of C3 into C3a and C3b. C3b associates with the C3 convertase to form the C5 convertase, resulting in the generation of C5a and C5b. Ultimately, C5b combines with complement proteins C6, C7, C8, and multiple C9 molecules to form the membrane attack complex (MAC), which directly induces endothelial cell lysis and promotes graft rejection (34). Complement fragments C3a and C5a serve as potent chemoattractants, facilitating immune cell infiltration, vasodilation, increased vascular permeability, and inflammation. Additionally, complement components such as C3 fragments play dual roles in promoting further B cell activation and antibody production. C3dg-coated antigens enhance B cell receptor (BCR) signaling via co-ligation with complement receptors, thereby amplifying T-cell independent-activation of B cells and perpetuating inflammation (35).

### Complement-independent mechanisms

Non-HLA antibodies can also mediate injury independent of complement system activation. This occurs predominantly via antibody interactions with Fc receptors (FcRs) expressed on various immune effector cells. Fcγ receptors (FcγR) on macrophages, granulocytes, and natural killer (NK) cells recognize the Fc portion of bound antibodies, activating immune effector functions such as antibody-dependent cellular cytotoxicity (ADCC), phagocytosis, and pro-inflammatory cytokine production (26). FcγRIIIA (CD16), notably expressed on NK cells, particularly mediates ADCC, contributing to graft cell injury in the absence of complement (14). Conversely, regulatory Fc receptors like FcγRIIB provide inhibitory signals limiting excessive immune activation, representing a critical balance mechanism in antibody-mediated immunity. Dysfunction or mutations in these regulatory pathways have been associated with heightened susceptibility to autoimmune and inflammatory diseases, further emphasizing their importance (36). Additionally, non-HLA antibodies may directly interact with their target antigens on endothelial cells, initiating downstream signaling cascades independent of Fc receptor engagement. An example of this mechanism is anti-AT1R antibodies, which function agonistically by activating AT1R expressed on endothelial cell surfaces. Activation can lead to cell proliferation and production of inflammatory cytokines, further contributing to graft injury and rejection (37, 38). The neonatal Fc receptor, a non-classical FcR expressed in many different tissues, may also contribute to the pathogenic potential of IgG antibodies by extending their half-life through pH-dependent interactions, thereby prolonging the inflammatory and damaging potential of pathogenic antibodies (39).

## Perlecan/LG3

Perlecan is a large heparan sulfate proteoglycan prominently located in the basement membranes of vascular and epithelial tissues. The C-terminal fragment of perlecan, known as LG3 (laminin-like globular domain 3), possesses notable immunogenicity and is typically embedded within the vascular basement membrane (12, 20). LG3 plays roles in vascular homeostasis, endothelial cell proliferation, and repair processes following vascular injury. Under pathological conditions, such as endothelial dysfunction or apoptosis, LG3 is enzymatically cleaved by activated cathepsin L from apoptotic endothelial cells and released into circulation within apoptotic exosome-like vesicles. These circulating LG3 fragments can induce an immune response, thereby serving as both biomarkers and active participants in vascular injury (40).

In kidney transplantation, ECM-derived antibodies, such as anti-LG3 antibodies have emerged as important mediators of transplant-associated vascular damage and graft dysfunction. Elevated anti-LG3 antibody titers correlate significantly with AMR, delayed graft function (DGF), and decreased long-term renal graft function (41, 42). Cardinal et al. (41) demonstrated that anti-LG3 antibodies, primarily of IgG1 and IgG3 subclasses, can potentiate IRI and exacerbate vascular rejection by promoting complement activation and vascular inflammation. These processes include characteristic features such as C4d deposition, peritubular capillary inflammation, neointimal hyperplasia, and obliterative vascular remodeling (43). Soulez et al. (40) further supported these findings, showing that increased circulating LG3 impedes endothelial repair mechanisms and promotes vascular remodeling, highlighting LG3’s active involvement in perpetuating vascular damage post-transplantation.

Pre-transplant anti-LG3 antibody status has also proven clinically relevant. In hypersensitized kidney transplant candidates, Riesco et al. (44) found that 52% had anti-LG3 antibodies. Notably, hypersensitized patients who developed AMR post-transplantation were frequently positive for anti-LG3 antibodies, underlining their association with heightened immunological risk independently of HLA-DSAs. Moreover, Carroll et al. (45) identified that dual positivity for anti-LG3 and anti-AT1R antibodies pre-transplantation significantly increased the risk for acute rejection characterized by severe alloimmune vascular injury, suggesting a synergistic interplay between these autoantibodies in transplantation outcomes.

Mechanistically, anti-LG3 antibodies develop following endothelial injury that may result from IRI, uremia, calcineurin inhibitor toxicity, or acute rejection episodes. LG3 fragments released from injured endothelial cells provoke autoantibody formation through immune mechanisms involving both innate and adaptive immunity. Interestingly, recent evidence suggests that memory B cells against LG3 are T-cell independent, but production of these antibodies requires essential T-cell assistance, further underscored by observed reductions in antibody titers following immunosuppressive therapies, such as calcineurin inhibitors (46). Moreover, studies by Juillard et al. (47) elucidated that LG3-containing apoptotic exosome-like vesicles activate innate immune pathways, including γδT cells and TLR-mediated signaling, leading to robust autoimmune responses in experimental models.

While anti-LG3 antibodies are predominantly identified post-transplantation, their pre-transplant levels, especially in younger patients, correlate inversely with age and are independent of traditional factors like DSAs, suggesting distinct immunological pathways of sensitization (43). Importantly, Mawad et al. (48) found that pre-transplant anti-LG3 antibodies notably increase the risk of DGF when kidneys are subjected to cold ischemic storage. Conversely, the use of hypothermic perfusion appears to mitigate this risk, emphasizing how peri-transplant management can modulate the clinical significance of these antibodies.

Anti-LG3 antibodies are significantly involved in vascular injury and graft dysfunction post-kidney transplantation. They influence transplantation outcomes through complement-mediated mechanism with evident clinical implications in terms of rejection phenotypes and graft survival. Despite our current understanding, further research is necessary to fully elucidate their exact mechanisms of production, interaction with other immunological factors, and optimal management strategies to mitigate associated graft injuries.

## Antibodies against G protein-coupled receptors

Antibodies targeting GPCRs have gained attention as critical mediators of vascular injury and poor clinical outcomes in kidney transplantation. Two prominent GPCR targets of non-HLA antibodies are AT1R and ETAR, both of which are expressed on endothelial cells and vascular smooth muscle cells (20). These receptors physiologically regulate vascular tone, blood pressure, and sodium balance through their respective ligands, angiotensin II and endothelin-1 (ET-1). In the context of transplantation, antibodies against AT1R and ETAR have individually been linked to AMR, vascular inflammation, accelerated graft dysfunction, and reduced graft survival (49, 50). Due to their similarities in structure and function, anti-AT1R and anti-ETAR antibodies are frequently studied together, often measured concurrently in kidney transplant patient sera.

### Angiotensin type I receptor

AT1R is a GPCR involved in critical physiological functions, primarily regulating blood pressure and fluid balance through its ligand, angiotensin II. Activation of AT1R leads to vasoconstriction, stimulation of aldosterone secretion, and sodium reabsorption in the kidneys (51). AT1R is expressed in renal tissue, including endothelial cells, smooth muscle cells of the renal vasculature, mesangial cells, podocytes, proximal tubular epithelial cells, and interstitial cells (52, 53). Unlike HLA molecules, AT1R is not highly polymorphic and anti-AT1R antibodies are autoantibodies. Initially, these antibodies were seen in cases of preeclampsia where the antibodies were specific to the second extracellular AT1R loop (54). Additionally, these antibodies have a pathophysiological involvement in several other diseases, such as systemic sclerosis and cardiovascular pathologies (37, 54–56).

Anti-AT1R antibodies were first described and characterized by Dragun et al. (37) who reported their role in AMR, severe hypertension, and graft dysfunction in kidney transplantation. Subsequent studies further delineated their pathogenic role, associating anti-AT1R antibodies with various poor transplant outcomes such as vascular injury, graft dysfunction, AMR, and allograft loss (57, 58). However, results have not been universally consistent, with some recent studies failing to find significant associations (59–62).

A recent systematic review encompassing 4023 pediatric and adult kidney transplant recipients found that both pre- and post-transplant anti-AT1R-positive patients had significantly higher incidences of AMR and graft loss compared to antibody-negative recipients (63). Similar findings were echoed by Lefaucheur et al. (64) where kidney transplant recipients with anti-AT1R antibodies demonstrated a distinct AMR phenotype characterized by vascular inflammation without complement deposition, emphasizing complement-independent mechanisms.

Importantly, pre-transplant anti-AT1R antibody detection has emerged as a significant risk factor for acute rejection and reduced graft survival. Giral et al. (65) demonstrated that relatively low pre-transplant levels above 10 U/mL strongly predicted poor graft function and acute rejection. Lee et al. (66) also found associations between pre-transplant AT1R antibodies and the onset of acute rejection within the first year post-transplantation. Conversely, the study by Filiopoulos et al. (60) reported no substantial impact of anti-AT1R antibodies on impaired graft functions and patient survival, highlighting discrepancies and limitations in clinical interpretation. The pathogenicity of anti-AT1R antibodies may depend on IgG subclass composition rather than total antibody levels alone. Mizera et al. (67) demonstrated that the IgG3 anti-AT1R antibodies was significantly associated with AMR and showed better predictive performance than total anti-AT1R IgG, suggesting that subclass profiling may improve risk stratification.

The interplay between anti-AT1R antibodies and HLA-DSAs also influences clinical outcomes significantly. Studies have shown that the coexistence of anti-AT1R antibodies with HLA-DSAs further exacerbates graft damage, resulting in accelerated rejection and poorer graft survival compared to DSAs alone (50, 58, 68, 69). Taniguchi et al. (68) particularly noted accelerated graft loss when pre-transplant anti-AT1R antibodies were combined with post-transplant *de novo* DSAs.

The mechanisms underlying anti-AT1R antibody formation are not fully elucidated but likely involve both autoreactive and alloreactive responses. Known sensitizing events include prior transfusions, pregnancies, previous transplants, and tissue injury during infections, which expose cryptic self-antigens (70). Moreover, *de novo* anti-AT1R antibodies may develop post-transplantation due to increased AT1R expression following allograft damage, possibly influenced by genetic polymorphisms such as A1166C in the *AGTR1* gene, affecting receptor expression levels (71). AT1R genotyping of transplant patients is not routinely conducted in the clinic, so the influence of polymorphisms is not supported by strong evidence.

AT1R antibodies induce graft injury primarily through their agonistic action on AT1R, independent of complement activation despite their IgG1 and IgG3 subclasses. Upon binding to the second extracellular loop of AT1R, these antibodies chronically activate signaling pathways such as phospholipase C and protein kinase C, along with Ras/Raf/MEK/ERK cascades, leading to endothelial activation, inflammatory cytokine production, and procoagulant states (20, 30, 72). This process promotes MVI, thrombosis, vascular endothelial cell proliferation, vasoconstriction, and eventually vascular rejection and graft loss (37, 73). A recent mechanistic study demonstrated that anti-AT1R and anti-ETAR IgG antibodies isolated from sera of patients with kidney transplant vasculopathy activated the mTOR complexes via AT1R and ETAR in a PI3K-dependent and ERK-independent manner and impaired endothelial repair (38). In a pediatric cohort, the upregulation of adhesion molecules ICAM-1 and VCAM-1, were associated with patients with elevated circulating anti-AT1R and anti-ETAR antibodies (74). Since adhesion molecules facilitate the trans-endothelial migration of immune cells, their upregulation can be a sign of MVI due to these non-HLA antibodies. Notably, anti-AT1R antibodies can amplify immune responses by recruiting inflammatory cells to the graft, perpetuating endothelial injury and further AT1R expression, thus creating a vicious cycle of damage. The relationship between anti-AT1R antibodies and HLA antibodies is complex. Anti-AT1R antibodies may facilitate the formation of DSAs by promoting vascular injury and inflammation, thereby exposing alloantigens and amplifying humoral immune responses.

Pediatric studies have demonstrated that anti-AT1R antibodies are more prevalent in younger recipients and may present unique clinical challenges. Pearl et al. (75) highlighted increased antibody prevalence and significant early rejection risks among pediatric kidney transplant recipients. They noted distinct temporal associations, such as short-term declines in renal function and increased cytokines post-transplantation, as well as a longer-term association with the development of HLA-DSAs. Hesemann et al. (70) similarly identified early post-transplant formation of anti-AT1R antibodies in pediatric recipients, emphasizing the need for age-specific management strategies. Host immune genetics may also influence anti-AT1R antibody levels. Yantir et al. (76) reported significantly higher anti-AT1R antibody levels in pediatric compared with adult kidney transplant recipients and identified associations between antibody levels and cytokine gene polymorphisms, including TGF-β1 and IL-6 variants, although no direct relationship with rejection was observed.

Current literature emphasizes critical knowledge gaps regarding anti-AT1R antibodies. The precise pathogenic mechanisms standardized diagnostic thresholds, and clinical significance of antibody titers remain uncertain. Notably, different studies have applied varied cut-off values (ranging from 10 to 17 U/mL) to define antibody positivity, complicating clinical interpretation (68, 75). Moreover, there is insufficient robust evidence supporting routine screening or specific therapeutic interventions such as angiotensin receptor blockers (ARBs), mTOR inhibitors, or immunosuppressive adjustments based solely on anti-AT1R antibody detection.

### Endothelin A receptor

Another GPCR target of non-HLA antibodies associated with poor kidney transplant outcomes is ETAR. In physiological conditions, this receptor is activated by its ligand, ET-1, which is a peptide stemming from endothelial cells. Activation of ETAR by ET-1 leads to the regulation of vasoconstriction, cell growth, cell migration, and sodium balance (77). In contrast, prolonged activation of ETAR can lead to pathological conditions, such as hypertension, vascular injury, and fibrosis (78). ETAR is expressed in multiple renal compartments including vascular endothelial and smooth muscle cells, mesangial cells, and tubular epithelial cells (20). Expression levels can be influenced by both genetic factors and environmental stimuli like IRI and inflammation, which are common in the transplantation context (79). These stressors increase ETAR surface expression and facilitate its recognition by circulating antibodies.

Initially, increased titers of anti-AT1R antibodies and anti-ETAR antibodies were noted post-heart transplantation and were associated with enhancing the alloimmune response, cellular and humoral rejection, and micro vasculopathy (80). There has been a plethora of evidence for AT1R serving as an independent risk factor for AMR in kidney transplants, but anti-ETAR has more limited evidence. Although anti-ETAR and anti-AT1R antibodies often co-occur, multiple studies have demonstrated that anti-ETAR antibodies are independently associated with poor kidney transplant outcomes (49). For example, a large clinical study of 252 transplant recipients found that patients positive for anti-ETAR antibodies had a 5.84-fold higher risk of developing AMR compared to negative controls (81). Furthermore, the risk of AMR rose greatly when both anti-ETAR and HLA-DSA were present. Other studies have confirmed that anti-ETAR antibodies are linked to increased incidence of vascular rejection, reduced estimated eGFR, and higher graft loss within the first-year post-transplant (82).

Histopathological studies have provided additional support for these clinical associations. Renal biopsies from anti-ETAR–positive patients often display features of acute vascular rejection, such as arteritis and endothelial activation (20, 83). Notably, glomerular ETAR expression has been associated with AMR, and some patients with ETAR positivity lost their graft within 12 months, despite inconclusive associations with circulating antibody levels (9, 84). This supports the hypothesis that anti-ETAR antibodies may bind their targets during active rejection and thus evade detection in serum assays. Moreover, pediatric studies have shown that anti-ETAR antibody presence is linked with a significant (>30%) decline in eGFR and an upregulation of adhesion molecules like ICAM-1 and VCAM-1, which may precede histological damage (74, 75). However, a recent multicenter study found no significant predictive value for anti-ETAR antibodies in isolation when monitoring for rejection, underscoring the need for better patient stratification and biomarker validation (85).

Anti-ETAR antibodies, which are typically of the IgG1 subclass, recognize a specific peptide sequence in the receptor’s second extracellular loop (20). Mechanistically, anti-ETAR antibodies can induce graft injury through both complement-dependent and complement-independent pathways (49). In complement-dependent cases, these IgG1 antibodies can activate the classical complement cascade, resulting in C4d deposition in peritubular capillaries. Alternatively, anti-ETAR antibodies can act in a complement-independent fashion similar to anti-AT1R antibodies by mimicking ET-1 binding. This can lead to sustained receptor activation, ERK pathway signaling, and proinflammatory cytokine production (20, 49). More recently, novel mechanisms have been proposed. As mentioned earlier, Catar et al. (38) demonstrated that anti-ETAR antibodies may promote endothelial injury through activation of the PI3K/mTOR pathway and recruitment of β2-arrestin, independent of ERK signaling. These pathways may impair endothelial repair and contribute to transplant vasculopathy, further supporting the rationale for using mTOR inhibitors as a targeted therapeutic strategy in this context.

When monitoring both anti-AT1R and anti-ETAR antibody levels, it was found that higher graft loss was experienced when elevated levels of these titers were found (86). A similar mechanism is suggested for the development of anti-ETAR antibodies as was suggested for anti-AT1R antibodies. The determinants may initially be hidden, however, present themselves during the injury and ultimately result in an autoimmune response (87). The presentation of the anti-ETAR antibodies may also be a secondary consequence to activation of the immune system or may possibly relate to acute rejection. Anti-ETAR antibodies may be preformed or arise *de novo* post-transplantation. One investigation found that nearly half of kidney transplant recipients were anti-ETAR positive before surgery (82). Whether preformed or *de novo*, their presence has consistently been associated with adverse clinical outcomes, including early AMR, progressive allograft dysfunction, and, in some cases, graft loss (75, 82, 84). The frequent co-occurrence with anti-AT1R antibodies raises questions about synergistic or additive pathogenic effects, though some studies have demonstrated that anti-ETAR antibodies can function independently to induce vascular inflammation and injury (38, 82).

Despite the growing body of evidence supporting a pathogenic role for anti-ETAR antibodies in kidney transplantation, significant knowledge gaps remain. It is currently unclear whether routine screening for these antibodies should be implemented pre- or post-transplantation, and universally accepted standardized thresholds for antibody positivity have yet to be defined. Furthermore, while mechanisms of anti-ETAR antibody-mediated injury have become increasingly understood, the clinical relevance of varying ETAR expression patterns in biopsy specimens still requires further validation.

## Major histocompatibility complex class I chain-related molecule A

MICA is a highly polymorphic, non-classical MHC molecule encoded within the MHC region on chromosome 6, adjacent to the classical HLA genes. MICA shares structural similarities with HLA class I molecules, but it does not associate with β2-microglobulin and has restricted distribution, predominantly expressed under stress conditions on endothelial cells, epithelial cells, fibroblasts, and dendritic cells (88, 89). MICA serves as a ligand for the activating NKG2D receptor on NK cells and cytotoxic T cells, bridging innate and adaptive immune responses during cellular stress or injury. In kidney tissues, MICA expression is notably upregulated under conditions of cellular stress, ischemia, and inflammation, making it a target for immune recognition post-transplantation (89).

One of the first papers highlighting the clinical relevance of anti-MICA antibodies in kidney transplantation was by Zou et al. (90). They reported that pretransplant anti-MICA antibodies in recipients were associated with significantly lower graft survival rates compared to those without these antibodies (88.3% vs. 93.0% at one year). Importantly, among recipients with well-matched HLA kidneys, MICA sensitization further lowered graft survival (83.2% vs. 95.1%). Following this initial report, several other studies confirmed similar associations, showing lower graft survival in patients with pretransplant anti-MICA antibodies (91–93).

Despite these findings, early studies on MICA antibodies in transplantation had significant methodological limitations. Primarily, donor and recipient MICA genotyping were seldom performed, raising uncertainty about the specificity of detected anti-MICA antibodies. Consequently, it remained unclear if these antibodies were genuinely donor specific. Furthermore, early investigations yielded conflicting results where no significant adverse outcomes associated with anti-MICA antibodies were found (94, 95). Their cohorts showed comparable rates of acute rejection episodes, creatinine levels, and long-term graft survival between MICA antibody-positive and negative recipients, challenging the pathogenic significance of these antibodies.

More recent studies addressed the methodological shortcomings by performing detailed MICA genotyping and assessing HLA-DSAs. A study by Baranwal et al. (96) reported that donor-specific anti-MICA antibodies were strongly linked to AMR, supporting the pathogenic role of these antibodies in kidney transplantation. Further, Ming et al. (97) demonstrated that posttransplant anti-MICA antibodies negatively impacted graft survival and function over a six-year follow-up, reinforcing clinical concerns surrounding these antibodies. Lee et al. (98) provided evidence of anti-MICA antibodies correlating significantly with chronic graft damage, specifically interstitial fibrosis and tubular atrophy, emphasizing their potential as biomarkers for chronic allograft dysfunction. This was further supported by a large multicenter study demonstrating that donor-recipient mismatches at the MICA locus and the presence of donor-specific anti-MICA antibodies substantially increased AMR and graft loss risks, particularly in synergy with classical HLA-DSA (99). Interactions between anti-MICA antibodies and classical HLA systems have been increasingly recognized. Carapito et al. (99) illustrated that combined anti-MICA and classical HLA-DSA significantly amplified the risk of graft loss, suggesting a cooperative pathogenic effect.

The mechanisms behind anti-MICA antibody formation involve sensitizing events common to HLA allosensitization, such as blood transfusions, pregnancy, or previous transplantation (20). However, anti-MICA antibodies may also form uniquely through tissue-specific cellular stress or injury events during transplantation (100). Both preformed and *de novo* development of anti-MICA antibodies have been documented, though *de novo* antibodies, appearing post-transplant, may pose greater risks due to active ongoing graft-directed immune responses (99).

Although increasing evidence links anti-MICA antibodies to poor transplant outcomes, several important limitations and gaps in understanding remain. Key among these is the precise pathogenic role and clinical utility of monitoring anti-MICA antibodies, given conflicting results and variable testing methods. The clinical benefit of routine pre- or post-transplant MICA genotyping and antibody testing remains unclear.

## Vimentin

Previous studies have linked anti-vimentin antibodies to cardiac allograft vasculopathy in patients who received a cardiac transplantation, but they also have an association with renal graft dysfunction (101). Vimentin is a type III intermediate filament cytoskeletal protein expressed in various cell types including endothelial cells (102). Under normal physiological conditions, it resides intracellularly. However, in the context of cellular injury, apoptosis, or inflammation, such as that occurring during allograft rejection or IRI, vimentin can become aberrantly expressed on the cell surface or released into circulation (103). This aberrant exposure allows the recipient immune system to recognize vimentin as a target, triggering the antibody production. Additionally, a pro-inflammatory environment can reduce regulatory cytokines like IL-10 and increase Th2-skewing cytokines such as IL-4 to enhance anti-vimentin production (104).

Carter et al. (102) reported that among patients who experienced graft failure, 44% developed *de novo* anti-vimentin antibodies during graft failure with titers rising within the first three months post-transplant and remaining persistently elevated. Notably, these patients had a 50% incidence of steroid-resistant vascular rejection, compared to <5% in the general transplant population. Following kidney transplantation, anti-vimentin antibodies has been detected in rats, where *de novo* IgG against vimentin were observed within 4–12 weeks post-transplantation (105).

The positive association between anti-vimentin antibodies and histological findings of interstitial fibrosis and tubular atrophy (IFTA) has been established in several studies (103–107). Chronic antibody-mediated injury is known to play an important role in IFTA, which can harm long-term renal graft survival. Lopez et al. (106) conducted a retrospective analysis investigating 97 transplant recipient sera and found that pre-transplant anti-vimentin antibody concentration over 15 μg/mL was a significant predictor of IFTA, but not graft loss. Elevated titers were especially pronounced in those who also had anti-HLA DQ2 donor-specific antibodies, suggesting a synergistic effect between anti-vimentin and anti-HLA antibodies on renal transplant outcomes (102, 105). Post-transplant elevated levels of the IgG subtype, but not IgM, were associated with IFTA and graft dysfunction in renal transplant patients, highlighting the role of anti-vimentin antibody isotypes (107).

It is still unclear whether these antibodies are directly pathogenic or simply markers of underlying tissue injury and inflammation. The precise mechanisms of injury are not fully understood. Additionally, there is limited information on the temporal relationship between anti-vimentin antibody appearance and the onset of specific histopathological changes, making it difficult to determine their predictive value. Current clinical studies are also limited by small sample sizes and heterogeneous patient populations.

## Collagen and fibronectin

Collagen and fibronectin are extracellular matrix proteins that serve as structural components of the glomerular basement membrane (GBM) and interstitial matrix. Similar to anti-LG3 antibodies, antibodies directed against collagen and fibronectin are ECM-derived antibodies. Type IV collagen, a network-forming collagen, is a principal element of basement membranes, whereas types I and III belong to the fibrillar collagen family and are more prevalent in the renal interstitium and vasculature (108, 109). Fibronectin, another matrix glycoprotein, plays roles in cell adhesion, wound healing, and tissue repair. Under normal physiological conditions, these proteins are sequestered from immune surveillance. However, following ischemic or inflammatory injury, they may become exposed, triggering autoreactive immune responses (20).

In kidney transplant recipients, immune responses to collagen IV and fibronectin have been implicated in the development of transplant glomerulopathy (TG), a hallmark of chronic rejection characterized by GBM duplication. Angaswamy et al. (110) found that antibodies to collagen IV and fibronectin post-transplant were associated with a 22-fold increased risk of developing TG. These antibodies were of IgG and IgM isotypes and were often accompanied by T cell responses skewed toward IFN-γ and IL-17 secretion, along with reduced IL-10 expression, indicating a loss of peripheral tolerance. Importantly, this immune activation was not contingent upon the presence of HLA-DSAs, suggesting that collagen and fibronectin antibodies can arise independently and still contribute to graft injury.

A similar pattern was observed in a pediatric transplant recipient cohort where *de novo* antibodies targeting collagen IV, fibronectin, and AT1R after transplantation were identified, with titers highest for AT1R but still notable for the matrix antigens (70). These antibodies emerged in the absence of pre-transplant inflammation and did not correlate with immediate clinical outcomes, though their long-term significance remains to be determined. Supporting this, Nair et al. (111) reported no statistically significant association between anti-collagen IV or fibronectin antibodies and chronic immune injury at 1- or 2-years post-transplant, though their study was limited by sample size and did not account for synergistic effects with other antibodies.

Anti-collagen I and III antibodies have recently gained attention for their potential diagnostic and prognostic value in AMR. In a study by Park et al. (112), patients with AMR had significantly higher titers of anti-collagen I and III compared to TCMR or no-rejection controls. Furthermore, each standard deviation increase in antibody level was associated with a significantly elevated risk of death-censored graft failure. These findings propose anti-collagen I and III as potential non-HLA biomarkers for AMR, especially in patients lacking HLA-DSA.

Broader cohort analyses have supported these associations. Senev et al. (113) used a high-throughput Luminex bead assay testing for 82 non-HLA antibodies and found that pre-transplant antibodies to collagen III were independently associated with the histological features of AMR. Similarly, another study revealed that renal transplant recipients who were endothelial cell crossmatch (ECXM) positive had increased expression of anti-collagen and anti-fibronectin antibodies. These antibodies clustered with other pro-fibrotic and inflammatory markers, suggesting their involvement in the ongoing allograft injury process, potentially triggered by initial tissue damage (114).

Mechanistically, the development of antibodies to self-antigens like collagen and fibronectin may stem from initial ischemia-reperfusion or alloimmune injury that exposes cryptic epitopes. These antigens may also be packaged in donor-derived exosomes, as demonstrated by Itabashi et al. (115), who identified collagen IV and fibronectin within circulating exosomes alongside immunomodulatory molecules in murine models of transplant rejection. These exosomes may prime recipient immunity toward self-antigen reactivity and promote chronic injury via both direct and indirect immune activation.

Anti-collagen and anti-fibronectin antibodies represent an expanding category of non-HLA antibodies associated with chronic allograft injury and AMR. While antibodies against type IV collagen and fibronectin have been directly implicated in TG, type I and III collagen antibodies are emerging as markers of AMR and poor graft survival. These responses may occur independently of HLA-DSA and involve complex T cell cytokine shifts. However, their predictive value, threshold titers, and synergy with other allo- and autoantibodies remain to be clarified.

## Natural antibodies

Nabs are polyreactive IgG and IgM antibodies with low affinity but high avidity, capable of recognizing a broad range of self and non-self antigens, including apoptotic cells, DNA, and oxidation-related epitopes such as malondialdehyde (MDA) (116). Traditionally produced by innate-like B1 cells and marginal zone B cells, Nabs serve as a bridge between innate and adaptive immunity and are present at low levels in healthy individuals (29). However, emerging evidence implicates their pathogenic potential in kidney transplantation, particularly in the context of rejection and graft dysfunction.

Multiple studies have shown that elevated Nabs, both preformed and *de novo*, are independently associated with adverse kidney transplant outcomes. In a study comparing patients with stable kidney grafts to those diagnosed with AMR, serum samples from the AMR group were found to contain polyreactive IgG antibodies that exhibited significantly greater reactivity toward an apoptotic cell model and enhanced complement-activating properties compared to those from the stable group (117). Following these initial findings, it was shown that pretransplant serum samples containing Nabs had an association with late graft loss by the same researchers (28). The pathogenic potential of Nabs has been reinforced in other cohorts. In a study of 635 transplant recipients, a 50% increase in post-transplant serum Nabs to MDA was associated with significantly worse long-term graft survival and higher Banff histologic scores for MVI, glomerulopathy, and C4d deposition (118). Multivariable analysis confirmed Nabs as an independent predictor of graft loss, even in the absence of DSA. Additionally, patients with both Nabs and DSA exhibited the poorest survival outcomes.

More recently, a large multicenter cohort of 980 kidney transplant recipients reported that elevated pretransplant IgG Nabs reactive to apoptotic cells were significantly associated with increased risks of graft loss, TCMR, and graft dysfunction, but there was no association with Nabs and AMR (119). These findings may highlight the limitations of the conventional histological scoring system, further exacerbated by inter-center variability in interpretation. Moreover, patients with high pre-transplant Nabs who subsequently developed post-transplant Nabs experienced markedly higher risks of TCMR and mixed rejection (117). Notably, the co-presence of Nabs and HLA-DSAs further amplified these risks.

Mechanistically, the contribution of Nabs to graft injury remains unclear. Some reports suggest complement-dependent effects of these IgG antibodies with the presence of C4d deposition (117). However, the cohort by See et al. (119), found no correlation between Nabs and AMR or C4d, indicating possible complement-independent pathways. One hypothesis is that Nabs bind to neoepitopes on apoptotic or damaged endothelial cells and trigger downstream immune responses without classical complement activation, similar to anti-AT1R antibodies. Furthermore, transcriptomic analyses have identified innate B cell subsets within kidney allografts during AMR, suggesting that polyreactive B1 cells may locally produce Nabs that perpetuate inflammation and graft rejection (29). The interplay between humoral and cellular immunity is also noteworthy. Van Besouw et al. (120) observed a positive correlation between circulating Nabs and donor-reactive IFN-γ–producing T cells in long-term transplant recipients, particularly among those who had experienced rejection, supporting a model in which Nabs may amplify or co-regulate T cell responses. Importantly, Nabs often emerge in patients with extended-criteria donor organs or prior sensitizing events, although they do not always correlate with DSA, suggesting distinct pathways of sensitization (118, 119).

While intravenous immunoglobulin (IVIg) therapy has shown partial modulation of Nab levels in some contexts, the impact of immunosuppressive regimens on Nab generation remains unclear (120). Current evidence suggests that Nabs may act both as active mediators of injury and as biomarkers indicating heightened immune activation or tissue stress. The clinical utility of measuring Nabs is limited by a lack of standardized assays, undefined threshold levels for risk stratification, and minimal integration into current transplant immunologic workups. The immunological triggers for Nab development, including whether they are purely innate or shaped by prior sensitization or tissue damage, also require clarification. Finally, the role of specific Nab targets, such as MDA and apoptotic cells, in influencing transplant outcomes remains underexplored.

## Glutathione S-transferase theta 1

GSTT1 is an enzyme belonging to the glutathione S-transferase family, a superfamily of proteins crucial for detoxifying endogenous and exogenous toxic compounds by conjugating them with glutathione (121). GSTT1 is expressed in various tissues, including hepatocytes and renal tubular cells (122). Anti-GSTT1 antibodies have increasingly been implicated in AMR and graft dysfunction in kidney transplants in recent years. Although these antibodies were initially involved in liver transplants due to the high GSTT1 expression in the liver, Aguilera et al. (123) first reported that anti-GSTT1 antibodies were linked to chronic AMR in kidney recipients without detectable HLA-DSAs. In this cohort, antibodies emerged between 32 and 60 months post-transplant and were associated with both C4d deposition and chronic AMR. Further supporting these findings, another study reported anti-GSTT1 antibodies in 31.6% of patients with AMR and C4d deposition, which is significantly higher than the 7.7% observed in the control group with graft dysfunction and negative C4d (124). Shortly after, Akgul et al. (125) observed no direct relationship between anti-GSTT1 antibodies and AMR, though antibodies were more prevalent in C4d-positive rejection episodes compared to stable graft function cases. These early studies produced mixed findings, complicating interpretation of the role of anti-GSTT1 antibodies in renal transplant outcomes.

More recently, these antibodies have gained increasing attention, particularly in the context of genetic mismatch, where GSTT1-null recipients receive GSTT1-positive grafts, leading to recognition of GSTT1 as a foreign antigen and the development of *de novo* anti-GSTT1 antibodies post-transplant (123, 124). Valencia Pereira et al. (126) frequently identified anti-GSTT1 antibodies in such mismatched kidney transplant recipients, though they did not observe significant effects on eGFR or graft survival in their cohort. In contrast, other studies have demonstrated strong correlations between high anti-GSTT1 antibody levels and AMR, independent of concurrent HLA-DSAs, suggesting these antibodies may serve as independent predictors of poor graft outcomes (21, 127). Obrișcă et al. (128) further showed that the GSTT1-null genotype strongly associates with anti-GSTT1 antibody formation, and both the genotype and antibody presence correlate with histological evidence of AMR. Anti-GSTT1 antibodies have been reported both independently and alongside HLA-DSAs, with several studies suggesting worse transplant outcomes when both are present (127).

Interestingly, in a study looking at hypersensitized patients waiting for a kidney transplant with cPRA > 98%, anti-GSTT1 antibodies had the highest prevalence (62%) in this cohort out of 23 non-HLA antibodies that were tested (44). Similar to other non-HLAs, these findings suggest the existence of sensitization events prior to transplantation, possibly due to previous transplants or blood transfusions. The pathogenic potential of anti-GSTT1 antibodies in graft injury likely involves injury through complement activation, endothelial injury, and subsequent inflammatory responses characteristic of AMR. These antibodies cause MVI and chronic tissue remodeling, leading to progressive graft dysfunction and eventual graft loss, as evidenced by histological analyses correlating antibody presence with classical signs of AMR (127, 128).

Currently, there remains uncertainty regarding the precise impact of anti-GSTT1 antibodies on graft outcomes and their direct pathogenic mechanisms independent of HLA antibodies. The different antibody positivity thresholds across studies complicates comparisons and undermines clinical utility. For instance, Comoli et al. (127) used an MFI threshold of 8031, whereas Obrișcă et al. (128) employed a significantly lower threshold of 3000 MFI. Specifically, the inconsistencies in studies reporting associations between anti-GSTT1 antibodies and graft survival necessitate a deeper understanding of antibody dynamics, antibody-antigen interactions, and factors influencing antibody pathogenicity.

## Detection methods

A range of detection methods are currently employed to detect non-HLA antibodies, each with distinct advantages and limitations. Cell-based assays, such as endothelial cell crossmatches, assess antibody binding to native antigens on donor-derived or cultured endothelial cells, providing physiologically relevant readouts but suffering from cellular heterogeneity and potential confounding by residual HLA expression. Advances using CRISPR-engineered glomerular endothelial cell lines lacking HLA expression aim to improve assay specificity by isolating non-HLA antibody reactivity (32). This model has shown clinical promise and has been used in a cohort of kidney transplant recipients for risk stratification, although multicenter validation is still required before broader adoption. Enzyme-linked immunosorbent assays (ELISA) are widely used solid-phase methods that enable quantitative detection of individual antibody specificities with relatively high specificity and accessibility. However, antigen immobilization may alter protein conformation, particularly for structurally complex targets such as GPCRs, potentially limiting detection of clinically relevant antibodies. In contrast, Luminex-based multiplex assays allow simultaneous screening of multiple antibody specificities using bead-based technology.

Accurate detection of non-HLA antibodies is increasingly recognized as essential for comprehensive immunological risk assessment in kidney transplantation. However, in contrast to the highly standardized approaches used for HLA antibody detection, non-HLA antibody testing remains limited by poor assay standardization, variability across platforms, and a lack of consensus guidelines for interpretation and clinical implementation. An essential aspect revealed by recent studies, including comparative analyses of commercial Luminex assays, is the profound heterogeneity and lack of analytical validity currently existing in the detection of non-HLA antibodies. A recent study reported considerable discrepancies in antibody prevalence and specificities in their cohort detected by two commercial assays, emphasizing significant analytical limitations (129). This heterogeneity likely arises from differences in protein sources for assay beads, contributing to conflicting study outcomes. Improving analytical validity through standardized, validated assays and reagents is critical before reliably comparing results across different cohorts or correlating findings with clinical outcomes. Additionally, thresholds defining positivity for non-HLA antibodies are inconsistent, typically set based on the standard deviation and the mean MFI, varying widely across studies. These discrepancies are problematic, especially considering demographic variations influencing antibody levels, such as gender, prior pregnancies, retransplantation status, age, and ethnicity. Large-scale, multicenter, prospective studies with diverse demographic representation, including healthy controls, are necessary to accurately establish clinically relevant thresholds.

## Total Non-HLA antibody burden and temporal dynamics

Beyond individual antibody specificities, the overall burden of non-HLA antibodies may reflect immunological risk in kidney transplantation. Broad profiling studies have shown that higher pre-transplant reactivity across multiple non-HLA antigens is associated with the development of histological features of AMR, indicating that cumulative sensitization may be more clinically relevant than individual antibody targets (113). In pediatric populations, the combined presence of HLA-DSAs and non-HLA antibodies has similarly been linked to increased microvascular inflammation and worse graft function, supporting a synergistic effect of total antibody load on graft injury (130).

However, the relationship between non-HLA antibody burden and clinical outcomes is not consistent across studies. In pediatric transplant candidates, non-HLA antibodies were present in approximately 53% of patients prior to transplantation, yet no significant association with AMR was observed over long-term follow-up (131). Antibody levels remained relatively stable over time, although patients who developed AMR exhibited more variability in their antibody profiles compared to those with non-AMR rejection. Similarly, recent work by Rajalingam et al. (132) demonstrated that both pre- and post-transplant global non-HLA antibody reactivity was not significantly associated with AMR. Interestingly, non-HLA antibody reactivity significantly declined following transplantation only in patients with functioning grafts, reflecting changes in immune activation over time. This reduction in overall antibody burden highlights that dynamic shifts in antibody profiles may better capture graft status and immune regulation than single time-point assessments.

Interpretation of non-HLA antibody burden is further complicated by observations in healthy populations. Studies using bead-based assays have shown that all individuals in low-risk healthy cohorts may test positive for non-HLA antibodies when manufacturer-defined thresholds are applied, raising concerns about assay specificity and overestimation of clinically relevant reactivity (133). Additionally, substantial heterogeneity in antibody reactivity has been observed across antigens and populations, with transplant candidates often exhibiting lower overall reactivity than healthy individuals (134). In particular, GPCR autoantibodies such as anti-AT1R are frequently detected in healthy individuals and may exert physiological or immunomodulatory effects under normal conditions (135). Accordingly, regulatory GPCR autoantibodies are increasingly recognized as active modulators of cellular signaling, immune and vascular homeostasis, with pathogenicity emerging primarily in the context of dysregulated titers, affinity, or signaling function. These findings underscore the need for standardized thresholds and population-specific reference ranges.

Collectively, these data suggest that while total non-HLA antibody burden may contribute to immunological risk, its clinical relevance is highly context dependent. Increasingly, evidence supports a model in which temporal changes in antibody levels and overall reactivity provide more meaningful insight into graft injury and immune activation than static measurements alone.

## Therapeutic avenues

Currently, there are no standard protocols or FDA-approved treatments for renal graft injury attributed to harmful non-HLA antibodies (26). This is likely the case because of the inconsistent associations between non-HLA antibodies and clinical presentations of graft injury, unlike the stronger associations with HLA-DSAs. Chronic and active AMR is a major cause of graft loss, including AMR related to non-HLA antibodies. Current strategies for treating AMR associated with non-HLA antibodies largely mirror those used for HLA-DSA-mediated AMR because of the similar effector antibody mechanisms leading to graft injury. Its management largely remains up to expert clinicians on a case-by-case basis, which commonly involves plasma exchange (PLEX), IVIg, corticosteroids, rituximab, proteosome inhibitors, and complement inhibitors (136–139). There is some clinical evidence supporting these therapies to treat AMR related to non-HLA antibodies, as reviewed by Kardol-Hoefnagel and Otten (140). However, these are based on single-center or observational studies, highlighting the critical need for more robust clinical trials to guide effective AMR management.

New therapeutic avenues also target key inflammatory pathways implicated in AMR. One promising avenue is IL-6 inhibition. IL-6 plays a pivotal role in plasma cell survival and antibody production. IL-6 inhibitors, including tocilizumab and clazakizumab, have demonstrated preliminary efficacy in reducing HLA-DSA titers and stabilizing renal function in patients resistant to conventional therapy (141–143). Early trials have shown promising outcomes, notably in reducing MVI and endothelial cell injury. Further robust clinical trials are ongoing to confirm the utility of these agents in routine clinical practice. Complement inhibition represents another promising therapeutic pathway. Agents such as eculizumab, which inhibits the terminal complement component C5, have shown efficacy in preventing early AMR episodes post-transplantation (144). However, long-term benefit remains inconsistent, particularly in patients with persistent high-level HLA-DSAs. Newer agents like C1 esterase inhibitors offer broader complement inhibition through the classical and lectin pathways, potentially addressing both early and late AMR stages more comprehensively (145, 146). Additionally, imlifidase, an IgG-degrading enzyme, has emerged as a unique strategy, rapidly reducing antibody levels by enzymatic cleavage. Clinical trials have demonstrated that imlifidase rapidly eliminates HLA antibodies, permitting transplantation in highly sensitized patients (147, 148). Although imlifidase treatment has not been extensively studied in the context of non-HLA antibodies, it has shown promise treating a case of anti-MICA antibodies (149). However, its application is restricted by rapid antibody rebound post-treatment, necessitating supplementary immunosuppressive therapies to prevent antibody resurgence (26). More recently, anti-CD38 monoclonal antibodies such as daratumumab have emerged as promising therapies by directly targeting antibody-secreting plasma cells and NK cells. Emerging clinical data suggest these agents can reduce donor-specific antibodies and improve histological features of AMR, although responses may be transient and variable (150).

Anti-AT1R antibodies are well-studied with studies reporting clinical cases of anti-AT1R specific AMR without the contribution of HLA-DSAs (63, 64). Standard management includes PLEX and IVIg in addition to AT1R receptor blockers, such as losartan or candesartan. Clinical studies have shown that this combination notably reduces vascular inflammation and subsequent tissue damage (37, 151). In a pediatric patient, losartan specifically has been associated with decreased tissue factor expression and improved vascular outcomes in graft biopsies (152). This receptor blockade has the potential to reduce antibody-mediated receptor activation and exert beneficial immunomodulatory effects. Prophylactic administration of AT1R blockers in patients identified pre-transplantation as high-risk due to elevated AT1R antibodies has shown similar rejection and graft survival outcomes compared to patients with low anti-AT1R antibodies, although larger randomized controlled trials are necessary to consolidate these findings (153). Current recommendations advise monitoring AT1R antibody levels in high-risk individuals pre- and post-transplantation to guide individualized therapeutic interventions (63).

Similarly, anti-ETAR antibodies also adversely affect renal graft survival. ETAR antagonists such as ambrisentan, bosentan, and macitentan have been studied as potential therapies. Animal studies demonstrate that selective ETAR antagonists significantly improve graft survival, creatinine clearance, and reduce chronic rejection indicators, suggesting promising translational potential (49). Limited clinical data support these findings, highlighting an area for focused future clinical trials.

Current therapeutic strategies against non-HLA antibodies in AMR involve repurposed approaches used against HLA-related rejection, highlighting a significant knowledge gap regarding specific non-HLA targets. Promising therapeutic agents such as IL-6 inhibitors, imlifidase, complement inhibitors, and anti-CD38 monoclonal antibodies are under investigation, potentially transforming future treatment protocols. Despite progress, limitations such as antibody rebound, incomplete B-cell depletion, and off-target immune suppression persist, necessitating combined therapeutic strategies and precise patient stratification. Future research should prioritize elucidating distinct mechanistic pathways of non-HLA antibodies to enable targeted interventions, supported by rigorous randomized clinical trials to establish clear guidelines and improve transplant outcomes.

## Conclusion

Non-HLA antibodies represent a diverse and complex group of immune mediators implicated in AMR and graft dysfunction in the context of kidney transplantation, particularly in cases where HLA donor-specific antibodies are absent. This review highlights key non-HLA targets, including anti-AT1R, anti-ETAR, anti-LG3, anti-vimentin, anti-MICA, anti-collagen, anti-fibronectin, natural antibodies, and anti-GSTT1, each associated with distinct biological functions and mechanisms of injury. Despite growing evidence linking certain non-HLA antibodies to adverse transplant outcomes, inconsistencies across studies remain prominent, driven in part by heterogeneity in detection methods, variability in patient populations, and a lack of standardized thresholds for positivity. These challenges underscore the need for cautious interpretation and limit the immediate clinical applicability of non-HLA antibody testing.

Moving forward, the diverse localization and function of non-HLA antigens ranging from membrane-bound receptors and intracellular proteins to extracellular matrix proteins warrant individual investigations into their clinical significance and pathophysiological mechanisms. Multiplex assays offer convenience but may obscure critical mechanistic insights. Thus, separate targeted investigations should employ mechanistic studies involving *in-vitro* and animal models to unravel sensitization processes, specific effector functions of antibodies based on inflammatory cellular pathways, and therapeutic implications. Epitope-level characterization will further refine our understanding of antibody specificity and support the development of more precise diagnostic assays and targeted therapies. Ultimately, large-scale, multicenter prospective studies with standardized methodologies are essential to validate clinical relevance, improve risk stratification, and translate these findings into meaningful advances in transplant diagnostics and personalized treatment strategies to improve long-term kidney transplantation success.
